# Opening Pandora's box: cause and impact of errors on plant pigment studies

**DOI:** 10.3389/fpls.2015.00148

**Published:** 2015-03-12

**Authors:** Beatriz Fernández-Marín, Unai Artetxe, Oihana Barrutia, Raquel Esteban, Antonio Hernández, José I. García-Plazaola

**Affiliations:** ^1^Institute of Botany and Center for Molecular Bioscience Innsbruck, University of InnsbruckInnsbruck, Austria; ^2^Department of Plant Biology and Ecology, University of the Basque Country (UPV/EHU)Bilbao, Spain; ^3^Institute of Agrobiotechnology, IdAB-CSIC-UPNA-Government of NavarraPamplona, Spain

**Keywords:** HPLC, plant pigments, erroneous data, reviewing process, chlorophyll, carotenoids, *Arabidopsis*

## Too many errors in scientific publications, too many in plant sciences, too

Today, there is an astonishing volume of scientific information available for researchers, which can be easily accessed through powerful search tools. Yet, the question now is whether all this vast amount of information is reliable. In this sense, a “bad science” controversy arose recently when many Open Access (OA) journals (more than a half) published a false, error-ridden paper, which had been submitted in order to test the publishing ethics of these journals (Bohannon, 2013). This fake article was published mainly by fraudulent journals, but it was also accepted by a number of OA journals of renowned publishers with peer-review systems. The failure to reject an article full of errors revealed that the system's gearbox is leaking somewhere. The carelessness of peer-reviews in a number of OA journals has opened a Pandora's Box, and what is more disconcerting, nobody can guarantee that it could not also affect regular journals (non OA). Traditionally, it has been assumed that scientific journals should detect and correct all these failings through the peer review before publication. Regrettably, as we show in this communication, the system is far from being perfect (Pulverer, 2010; Székely et al., 2014).

Whilst the detection of laboratory errors is an issue of great attention in medicine (Bonini et al., 2002; Carraro and Plebani, 2007; Hammerling, 2012), in experimental science, it does not seem to be a crucial task. However, we were aware of this concern when we performed a literature compilation with the aim of providing a comprehensive evaluation of the responses of photosynthetic pigment composition to environmental conditions (Esteban et al., 2015). In this survey, we compiled data from 525 papers from the last 21 years (1991–2011). After carrying out a critical analysis of the data, a considerable number of papers, 96 out of 525, were found to have data out of range, errors and inconsistencies in at least one of the parameters reported. In order to detect these errors, we used as an initial screening tool three standard deviations from the mean. Data outside this interval were subsequently examined (Osborne and Overbay, 2004), in order to identify whether errors had arisen from the inherent variability of the data or from mistakes in the data (for a detailed description see Esteban et al., 2015). We decided to carry out an in-depth analysis of these controversial data, establishing a new background for our study. In this sense, data was re-evaluated and classified on the basis of the type of error: I, II, and III. Error I (*n* = 46) includes those articles containing values out of range (outside ±3 standard deviation from the mean), likely due to pre-analytical and analytical flaws. Error II (*n* = 37) refers to the presence of wrong values (most of them higher than 1000-fold reference values), most likely caused by post-analytical errors. Error III (*n* = 13) includes those articles with mistaken units, probably included during the final phase of publishing and editing.

Discarding the non-ethical manipulation of data, errors may occur as a result of experimental (analytical or methodological), mathematical or editing errors. For the case studied here (pigment determinations), we have identified several potential sources of error, which may occur at any stage of the research process: (i) Methodological errors during **pre-analytical and analytical phase**: inappropriate specimen/sample collection and preservation, labeling errors, wrong biomass/leaf area measurement, incomplete extraction, malfunction of instruments, incorrect compound identification, pipetting errors, etc. (ii) Data analysis errors in the **post-analytical phase**: handling of mathematics in spreadsheets, mistakes in the preparation of graphics or tables, improper data entry, and failure in reporting; (iii) **Publishing and editing** errors: confusion in units, typing errors such as Latin instead of Greek letters or errors in graph scales (see Table S1 for complete list of errors, tips and solutions). The data published in regular papers do not allow an assessment to be made of which of these aforementioned error sources is the cause of the mistakes. However, in other fields, such as analytical medicine, in which traceability is easier thanks to the application of quality assurance protocols, most errors occur during the pre- and post-analytical phases (Hammerling, 2012).

## How, where and when these errors appear

**Figure 1:**
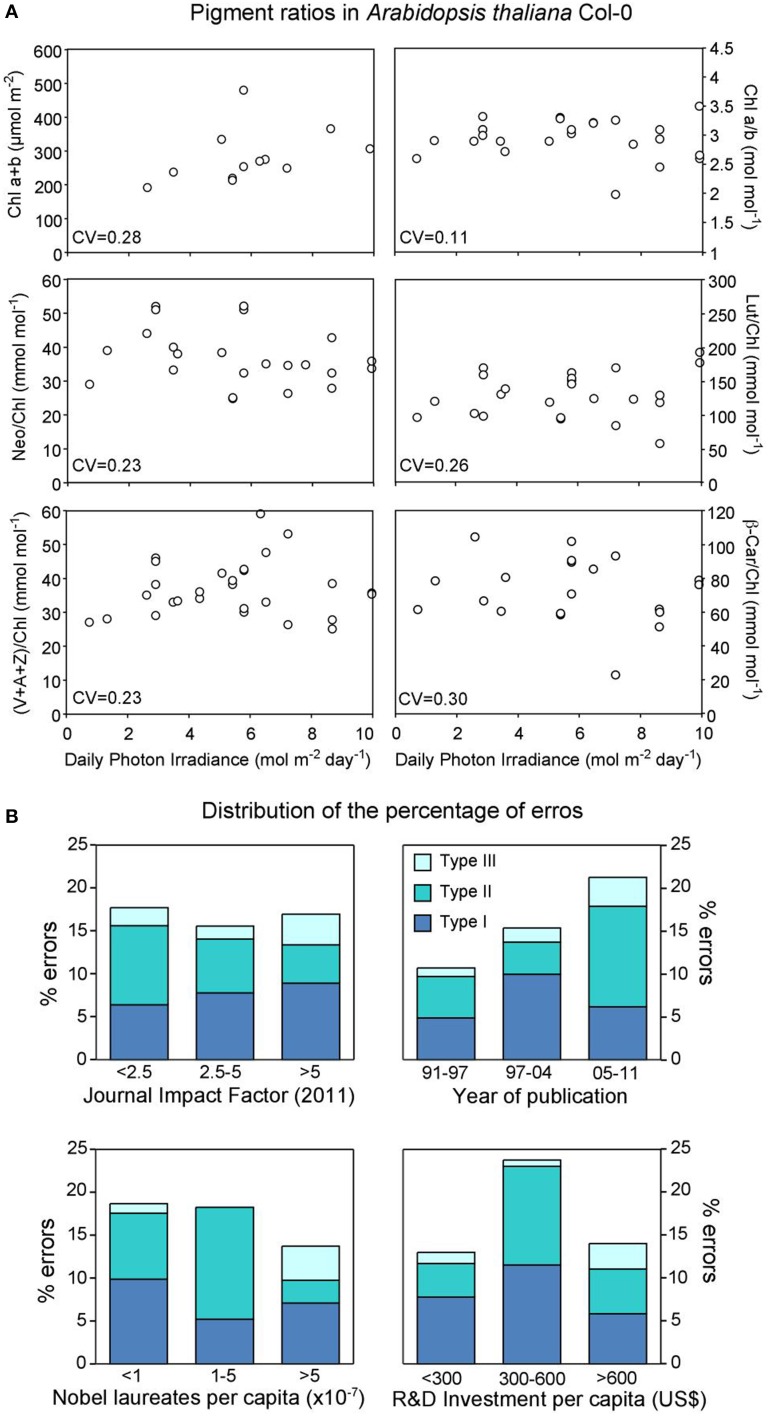
**(A)** Relationship between growth irradiance and pigment ratios in *Arabidopsis thaliana* Col-0. Coefficients of variation (CV, defined as the ratio of the standard deviation to the mean) are shown for each parameter. For data sources see Esteban et al. (2015). Abbreviations used: A, antheraxanthin; Chl, chlorophyll; Lut, lutein; Neo, neoxanthin; β-Car, β-carotene; V, violaxanthin; Z, zeaxanthin. **(B)** Relationship between the frequency of error types and: Impact factor of the journal, year of publication, number of Nobel Laureates per capita and research and development investment per capita. The frequency of errors is expressed as percentage within each class out of the 525 papers evaluated in Esteban et al. (2015).

As an example of the inaccuracy of pigment measurements, we have performed an analysis of data published on pigment composition in the model plant *Arabidopsis thaliana* ecotype Columbia including all the available literature published in the major journals in this field during the period 1991–2011 (see Esteban et al., 2015 for details). It is assumed that plants cultivated under similar conditions in different laboratories should not differ greatly in their biochemical attributes; otherwise *A. thaliana* would not be a good “model species.” To reduce the noise we considered only pigment ratios that are supposed to be much more stable than absolute concentrations. However, as is shown in Figure 1A, when pigment composition was plotted against cumulative daily irradiance, a factor that has been described as the main determinant of chlorophyll and carotenoid contents (Niinemets et al., 2003), no pattern of response was observed. Furthermore, it varied greatly (5-fold for some parameters). As it is unlikely that these ranges of variation represent true differences in pigment composition (carotenoids and chlorophylls in green tissues are bound to proteins with their strongly regulated and interdependent proportions and concentrations), they are probably a reflection of the lack of methodological accuracy probably affecting both pigment analysis itself and imprecisions regarding growing procedures or their descriptions throughout the manuscripts.

In general, it seems reasonable to consider that these errors and this lack of accuracy may stem from the careless application of protocols, from sample collection to data calculations and even final publication. In this sense one might expect that the scientific tradition of a country or the quality of the journal in which the research was performed would contribute to filtering out inconsistent results. In order to disentangle the influence of this kind of factors on the frequency of errors we have described the following variables for each discarded reference: (i) The journals' impact factor (IF) as an index of quality, (ii) the year of publication, (iii) the scientific heritage of the authors' country, estimated by the number of Nobel laureates, and (iv) the countries' current investment in Research and Development (R&D) (Figure 1B). Interestingly, our analysis shows that, perhaps just by excluding the scientific tradition factor, the frequency of errors escapes these biases, being similar both in groups from countries that invest a lot in science and have a consolidated research tradition, and in those from emerging economies with low investments in R&D (Figure 1B). Furthermore, even the quality of the journal (measured by the impact factor) does not filter out unreliable results (Figure 1B). Even more striking is the fact that, despite the technical and methodological improvements that have been made during the last two decades, the frequency of errors detected in the present survey shows a clearly upward trend (from 12 to 22% over a 21-year period) (Figure 1B). Perhaps this is the consequence of the pressure exerted by the well-known “publish or perish” dilemma that forces the rapid and careless publication of data. It should be noted here that we have focused our study on numerical errors that affect the quantitative expression of a value. Therefore, we have not considered other types of errors (incorrect use of statistics, experimental design, conceptual error and discursive errors).

The question that arises now is whether this inaccuracy relates only to pigment determinations, or whether this is just the tip of the iceberg, it being indicative of a general malaise in plant science studies. Although some studies have analyzed statistical limitations and biases, as well as methodological and statistical errors in ecology and plant science (Grime and Sinclair, 1999; Ross and Sulev, 2000; Heinemann et al., 2002; Dieleman and Janssens, 2011; Curtis and Queenboroughn, 2012; Hulme et al., 2013; Mudge, 2013) very little has been done to detect and quantify the appearance of quantitative errors. So, without further analyses, it is, of course, impossible to respond to this essential question. However, it is noteworthy that one of the simplest determinations a plant biology lab can perform is pigment content (relatively robust molecules, present at high concentrations, can be directly measured thanks to their absorbance in the visible range, their study does not require investments in expensive equipment). Thus, finding these incongruences regarding the data reported for these parameters should cause a stir among the entire scientific community.

## Lessons for the future: “errare humanum est, sed in errare perseverare diabolicum.”

This is a Latin quote attributed to Seneca, which means “Anyone can err, but to persist in error is diabolical.” Two thousand years later, in the “omics” era, when model species such as *Arabidopsis* are used as genomic reference material, such a degree of inaccuracy in analytical determinations is unacceptable, and the search for solutions is a must for the entire community of plant researchers. What is in question is not the reputation of researchers but the quality, accuracy and reproducibility of the results. All the actors in the scientific chain of activity, from the lab to the publishing house (technicians, researchers, authors, reviewers, editors and even readers), should be involved in the job of detecting and removing errors. Among them, the role of researchers and reviewers is of particular importance for the detection of inconsistent values.

The detection of errors has not been a crucial task in most plant biology research groups. However, in view of the experience gained by the methods employed by analytical laboratories to enhance the quality of results (Smagunova et al., 2006; Su et al., 2011), the reliability of data can be substantially improved by the implementation of inter-laboratory comparison tests. For pigments, these proficiency tests should be based on standard reference plant photosynthetic material (the standards for calibrating HPLC are not in themselves a guarantee) together with the availability of reference values for “normal” ranges (as those reported in Esteban et al., 2015) of pigment contents.

In the publication process, reviewers are considered to be infallible gods, and they must guarantee the quality of the experimental work, adequate data interpretation and the removal of all inaccuracies before publication. However, reviewers are frequently overloaded with too many commitments, and are forced to review manuscripts at odd moments during the day. Paradoxically, in the research publication chain, reviewers are one of the essential links, but at the same time they are the only amateur component (in the sense that their work is not paid), and their contribution is frequently not sufficiently recognized. Aditionally, as Plant Science studies are becoming interdisciplinary, classical review process is very limited. The correction policies of some journals that allow the publication of an amendment note or even the re-publication of a corrected version of the whole article are commendable. In that sense, the role of the whole scientific community as critical readers becomes an inescapable duty. A stronger implication of editors and readers, together with the presentation of results in a traceable form would also contribute to reducing the number of quantitative results in doubt. Also the creation of databases about plant attributes, where it could be easy to check whether a measured parameter falls into the expected range of values or not, could help in the prevention of incorrectness. Thus, this work does not set out to be a Casus Belli, but what it does say is that we must all try harder. Luckily, in the theater of scientific publications, the same actors (we researchers) have to play different roles (authors, readers, reviewers and editors) and this unusual trait of our field of professional activity should warrant the easy implementation of policies of quality assurance. In conclusion, improvements in data correctness could be done in three directions: (i) careful check of all steps in data obtention, analysis and publication (details in Table S1), (ii) more open review system, with easy postpublication procedures, and (iii) generation of databases of plant attributes that could be used as reference.

### Conflict of interest statement

The authors declare that the research was conducted in the absence of any commercial or financial relationships that could be construed as a potential conflict of interest.

## Supplementary material

Table S1 **Summary of main error types in plant pigment analysis, tips to detect them and potential solutions to correct or prevent them**.
