# Supplementary material for: Opening Pandora's box: cause and impact of errors on plant pigment studies
Source: Front Plant Sci. 2015 Mar 12;6:148. doi: 10.3389/fpls.2015.00148 (PMC4357246; doi:10.3389/fpls.2015.00148)
Supplement: Table S1 — Summary of main error types in plant pigment analysis, tips to detect them and potential solutions to correct or prevent them. [file Table1.PDF]

Error Type (phase)	Error	How to detect?	How to solve?
**Methodological (pre-analytical and analytical phase)**	Bad sample collection	Noisy baseline. Degradation products. Incoherent replicates	Careful planning and selection of plant material: consider leaf orientation, injures and imperfections, day-time and season of collection, developmental stage…
	Bad sample preservation	Noisy baseline. Degradation products (pheophytin). High isomerization of carotenoids	Check storage cold-chain<br>Use silica gel for dry storage (in case of freeze-dried material)
	Labeling error	Unexpected results. Incoherent results	Double labeling<br>Easy labeling (brief and simple, better than complex codes)
	Wrong biomass/area estimation	Anomalous concentrations	Weight/measure area before freezing
	Incomplete extraction	Pellet remains green	Double or triple extraction. Adjust the extraction volume to the sample biomass. Adequate the polarity of the extraction medium to the water content of the sample
	Extract degradation	Noisy baseline. Degradation products (pheophytin). High isomerization of carotenoids	Avoid always light and heat. Buffer the pH of the extraction medium
	Pipetting errors	Anomalous concentrations. Incoherent replicates	Training and pipetting calibration
	Contamination	Anomalous concentrations. Presence of unexpected peaks	Careful handling
	Instrument error	Weird peaks. Noisy baseline	Maintenance and knowledge of HPLC troubleshooting
	Bad calibration	Anomalous concentrations	Periodic calibration
	Analytical inaccuracy	Low peak resolution	Wide-shaped unresolved peaks
	Incorrect pigment identification	Anomalous presence and/or concentrations	Use pigment standards. Spike the sample. Check identification of the pigment with LC-MS
**Data analysis (post-analytical phase)**	Error in spreadsheets	Anomalous concentrations	Double checking
	Error in data entering	Anomalous concentrations	Double checking
	Error in figure preparation	Anomalous concentrations	Double checking
**Publishing and editing**	Confusion in units	Anomalous concentrations	Careful proof correction<br>Comparison of data with realiable references
	Typing errors	Anomalous concentrations	Careful proof correction<br>Comparison of data with reliable references
